# Care episode retrieval: distributional semantic models for information retrieval in the clinical domain

**DOI:** 10.1186/1472-6947-15-S2-S2

**Published:** 2015-06-15

**Authors:** Hans Moen, Filip Ginter, Erwin Marsi, Laura-Maria Peltonen, Tapio Salakoski, Sanna Salanterä

**Affiliations:** 1 Department of Computer and Information Science, Norwegian University of Science and Technology, Sem Saelands vei 9, 7491 Trondheim, Norway; 2 Department of Information Technology, University of Turku, Joukahaisenkatu 3-5, 20520 Turku, Finland; 3 Department of Nursing Science, University of Turku, Lemminkäisenkatu 1, 20520 Turku, Finland; 4 Turku University Hospital, Kiinamyllynkatu 4-8, 20521 Turku, Finland; 5 Turku Centre for Computer Science (TUCS), Joukahaisenkatu 3-5, 20520 Turku, Finland

## Abstract

Patients' health related information is stored in *electronic health records *(EHRs) by health service providers. These records include sequential documentation of care episodes in the form of clinical notes. EHRs are used throughout the health care sector by professionals, administrators and patients, primarily for clinical purposes, but also for secondary purposes such as decision support and research. The vast amounts of information in EHR systems complicate information management and increase the risk of information overload. Therefore, clinicians and researchers need new tools to manage the information stored in the EHRs. A common use case is, given a - possibly unfinished - care episode, to retrieve the most similar care episodes among the records. This paper presents several methods for information retrieval, focusing on care episode retrieval, based on textual similarity, where similarity is measured through domain-specific modelling of the distributional semantics of words. Models include variants of *random indexing *and the semantic neural network model *word2vec*. Two novel methods are introduced that utilize the ICD-10 codes attached to care episodes to better induce domain-specificity in the semantic model. We report on experimental evaluation of care episode retrieval that circumvents the lack of human judgements regarding episode relevance. Results suggest that several of the methods proposed outperform a state-of-the art search engine (Lucene) on the retrieval task.

## Introduction

The development, adoption and implementation of health information technology, e.g. *electronic health record *(EHR) systems, is a strategic focus of health policies globally [1-4] and the amount of electronically documented health information is increasing exponentially as health records are becoming more and more computerised. The vast amounts of computerised health information complicate information management and increase the risk of information overload. At the same time, it creates opportunities for technological solutions to support health related and clinical decision making. For instance, the use of *natural language processing *(NLP) methods to facilitate researchers in discovering new knowledge to improve health and care.

**Figure 1:**
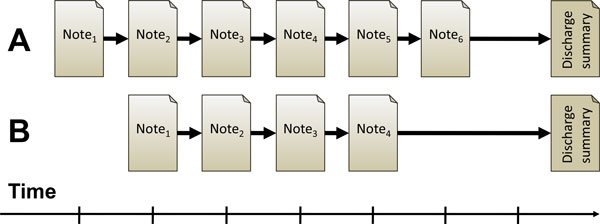
**Illustration of care episode retrieval**. The two care episodes (A and B) are composed of a number of individual clinical notes and a single discharge summary. Given an ongoing care episode (minus the discharge summary), the task is to retrieve other, similar care episodes.

EHRs are used throughout the health care sector by professionals, administrators and patients, primarily for clinical purposes, but also for secondary purposes such as decision support and research [5]. EHRs include structured and unstructured data, and they consist of a sequential collection of a patients health related information e.g. health history, allergies, medications, laboratory results and radiology images. Also, the different stages of a patient's clinical care are documented in the EHR as *clinical care notes*, which mainly consist of free text. A sequence of individual clinical care notes form a *care episode*, which is concluded by a discharge summary, as illustrated in Figure 1.

Information retrieval (IR) aims at retrieving and ranking documents from a large collection based on the information related needs of a user expressed in a search query [6]. IR has become a crucial technology for many organisations that deal with vast amounts of partly structured and unstructured (free text) data stored in electronic format, including hospitals and other health care providers. IR is an essential part of the clinical practice and clinicians, i.e. nurses and physicians search on the Internet for information, typically health literature, to solve clinical problems and for professional development [7]. Such online IR systems are associated with substantial improvements in clinicians decision making concerning clinical and health related problems [8,9]. To date, as the information in the EHRs is increasing, clinicians need new tools to manage the information. Therefore, IR from EHRs in general is a common and important task that, among other things, can support *Evidence-Based Practice *(EBP) through finding relevant care episodes and gathering sufficient evidence.

This paper focuses on the particular task of retrieving care episodes that are most similar to the sequence of clinical notes for a given patient, which we will call *care episode retrieval*. In conventional IR, the query typically consists of several keywords or a short phrase, while the retrievable units are typically documents. In contrast, in this work on care episode retrieval, the queries consist of the clinical notes contained in a care episode. The final discharge summaries for each care episode are assumed to be unavailable for constructing a query at retrieval time.

We envision a number of different use cases for a care episode retrieval system. Firstly, it could facilitate clinicians in care related decision making. For example, given a patient that is being treated in a hospital, an involved clinician may want to find previous patients that are similar in terms of their health history, symptoms or received treatments. Additional inputs from the clinician would enable the system to give more weight to keywords of particular interest within the care episodes, which would further be emphasized in the semantic similarity calculation during IR. This may support the clinician's care-related decision making when seeing what similar patients have received in terms of medication and treatment, what related issues such as bi-conditions or risks occurred, how other clinicians have described certain aspects, what clinical practice guidelines have been utilized, and so on. This relates to the principle of reasoning by analogy as used in textual case-based reasoning [10]. Secondly, when combined with systems for automatic summarization and trend detection, it could help health care managers to optimally allocate human resources with almost real time information concerning the overall situation on the unit for a specific follow-up period. Such a system could for example support managerial decision making with statistical information concerning care trends on the unit, adverse events and infections. Thirdly, it could facilitate knowledge discovery and research to improve care (cf. EBP). For instance, it could enable researchers to map or cluster similar care episodes to find common symptoms or conditions. In sum, care episode retrieval methods/systems hold large potential to improve documentation and care quality.

IR in the sense of searching text documents is closely related to NLP and is often considered a subfield of NLP. For example, stemming or lemmatization, in order to increase the likelihood of matches between terms in the query and a document, is a typical NLP task. From the perspective of NLP, care episode retrieval - and IR from EHRs in general - is a challenging task. It differs from general-purpose web search in that the vocabulary, the information needs and the queries of clinicians are highly specialised [11]. Clinical notes contain highly domain-specific terminology [12-14] and generic text processing resources are therefore often suboptimal or inadequate [15]. At the same time, development of dedicated clinical NLP tools and resources is often difficult and costly. For example, popular data-driven approaches to NLP are based on supervised learning, which requires substantial amounts of tailored training data, typically built through manual annotation by annotators who need both linguistic and clinical knowledge. Additionally, variations in the language and terminology used in sub-domains within and across health care organisations greatly limit the scope of applicability of such training data [12]. Moreover, resources are typically language-specific: most tools for processing English clinical text are of no use for our work on Finnish clinical text.

Recent work has shown that *distributional models of semantics*, induced in an unsupervised manner from large corpora of clinical and/or medical text, are well suited as a resource-light approach to capturing and representing domain-specific terminology [16-19]. The theoretical foundation for these models is the *distributional hypothesis *[20], stating that words with similar distributions in language - in the sense that they co-occur with overlapping sets of words - tend to have similar meanings. These models avoid most of the aforementioned problems associated with NLP resources. They do not involve the costly manual encoding of linguistic or clinical/medical knowledge by experts as required in knowledge-based approaches, nor do they involve equally costly investments in large-scale manual annotation and corpus construction as required for supervised learning. Instead, they can be constructed for any language or domain, as long as a reasonable amount of raw text in electronic format is available.

The work reported here investigates to what extent distributional models of semantics, built from a corpus of clinical text in a fully unsupervised manner, can be used to conduct care episode retrieval. The purpose of this study is to explore how a set of different distributional models perform in care episode retrieval, and also to determine how care episode similarity is best calculated. The models explored include several variants of *random indexing *and *word2vec*, methods which will be described in more detail in the 'Methods' section. These models allow us to compute the similarity between words, which in turn forms the basis for measuring similarity between texts such as individual clinical notes or larger care episodes. Several methods for computing textual similarity are proposed and experimentally tested in the task of care episode retrieval - being the main contribution of this paper.

It has been argued that clinical NLP should leverage existing knowledge resources such as knowledge bases about medications, treatments, diseases, symptoms and care plans, despite these not having been explicitly built for the purpose of clinical NLP [21]. Along these lines, a novel approach is presented here that utilizes the 10th revision of the International Classification of Diseases (ICD-10) [22] - a standardised tool of diagnostic codes for classifying diseases, labelled as meta-information to care episodes by clinicians - to better induce domain-specificity in the semantic model. Experimental results suggest that such models outperform a state-of-the art search engine (Lucene) on the task of care episode retrieval. Results also indicate that performance gain is achieved by most models when we utilize a list of health terms (cf. a health metathesaurus) for boosting term weights.

Apart from issues related to clinical terminology, another problem in care episode retrieval is the lack of benchmark data, such as the relevance scores produced by human judges commonly used for evaluation of IR systems. Although collections of care episodes may be available, producing gold standard similarity scores required for evaluation is costly. Another contribution of this paper is the proposal of evaluation procedures that circumvent the lack of human judgements regarding episode similarity. Two evaluation setups are used, one relying on ICD-10 codes attached to care episodes, and the other relying on textual semantic similarity between discharge summaries belonging to care episodes. Neither discharge summaries nor ICD-10 codes are used for constructing a query at retrieval time. This includes that sentences mentioning ICD-10 codes in free text are excluded from the query episodes. Despite our focus on the specific task of care episode retrieval, we hypothesize that the methods and models proposed here have the potential to increase performance of IR on clinical text in general.

This article extends earlier work published in Moen et al. [23]. New content includes the evaluation of various methods and setups on 40 instead of 20 query episodes, the introduction and evaluation of a new semantic model (W2V-ICD), and alternative ways of calculating care episode similarities.

The structure of this article is as follows. In the next section, 'Related work', we describe some related work. In the 'Task' section we describe in more detail the task of care episode retrieval, followed by a description of the data set of care episodes in the 'Data' section. The 'Methods' section presents six different distributional semantic models as well as two baselines. The 'Results' section reports the results of two experimental evaluations. The final two sections, 'Discussion' and 'Conclusion', are dedicated to discussion and conclusions respectively.

## Related work

With the issues of information overload in hospitals and the general need for research and improvements in clinical care, several IR systems have been developed specifically for health records and clinical text. Examples are the *Electronic Medical Record Search Engine *(EMERSE) [24], the *StarTracker *[25], the *Queriable Patient Inference Dossier *(QPID) [26], the *MorphoSaurus *[27], and the *CISearch *[28]. These software are used by clinicians and researchers to seek information from EHRs. Other IR systems used in multiple domains, including the health domain, is the open source search engine, or framework, *Apache Lucene *(Lucene) [29] and the Terrier search engine [30]. Some research has been published in relation to the use of these systems in the clinical domain [11,26,28,31-34]. However, research evaluating the effect of these tools and their impact on care and patient outcomes seems to be scarce. In this work Lucene is used as a baseline.

One challenge related to clinical NLP is the limited availability of such data, mainly due to its sensitivity. Thus, many IR/search solutions that are in use in various EHR systems today are often off-the-shelf generic IR tools, or unique to the corresponding EHR systems. In other words, the underlying IR methods are seldom subject to research on clinical IR. However, in recent years through shared tasks such as the TREC Medical Records track [35,36] and the ShARe/CLEF eHealth Evaluation Lab [37], clinical data is becoming increasingly accessible to a broader audience of researchers, thus research on clinical NLP and IR has gained some momentum. Existing work on IR for health records relies to a large extent on performing some type of query expansion, and possibly some bootstrapping, through the use of tailored information sources, or corpus-driven statistical methods. Limsopatham et al. [38] attempts to improve IR on health records by inferring implicit domain knowledge, mainly done through query expansion that relies on tailored domain-specific resources and information from other high-ranked documents. Zhu and Carterette [39,40] performs query expansion mainly through the use of more generic resources, including ICD-9, Medical Subject Headings (MeSH) and Wikipedia. They also explore the use of a negation detection tool for information exclusion (ConText [41]).

Distributional semantic models have enjoyed a steady popularity for quite some time, and have for instance recently gained a lot of interest with the introduction of the word2vec method by Mikolov et al. [42]. Such methods for inducing models of distributional semantics, in an unsupervised and language independent fashion, have shown to perform well at a range of NLP tasks, including more generic IR [43,6-47] and clinical IR for health records, see participants of the TREC Medical Records track [35,36]. Noteworthy, Koopman et al. [17] performs a comparison of several distributional models at clinical IR, including models built using the methods random indexing (RI) [48] and latent semantic analysis (LSA) [49]. There is no doubt that further research and evaluation of such methods would contribute to potential improvements in NLP, IR and information management in the clinical domain. One method that lacks proper evaluation in this domain is word2vec.

A promising direction in clinical NLP have been demonstrated through methods/systems that utilize various external knowledge resources, other than just the actual words in the query and target, either for performing query expansion [40], or in the textual similarity metric [50]. This is some of the underlying inspiration for a novel method contribution in this paper, one that relies on exploiting ICD-10 codes that has been labelled the care episodes. However, instead of using these for direct query expansion, they are utilized in the actual training phase of the semantic models.

Existing work on clinical IR that we are aware of relies on evaluation data where the queries are short search phrases. This differs from the task presented here, where the query is an entire care episode.

Diagnosis and treatment codes, such as ICD codes, are often applied at the end of the patient stay, or even after discharged from the hospital. Automatic labeling of care episodes with ICD codes has been the subject of a number of studies, e.g. [51,52]. Arguably this task is somewhat related to our task as far as the use of ICD codes for evaluation is concerned.

## Task

**Figure 2:**
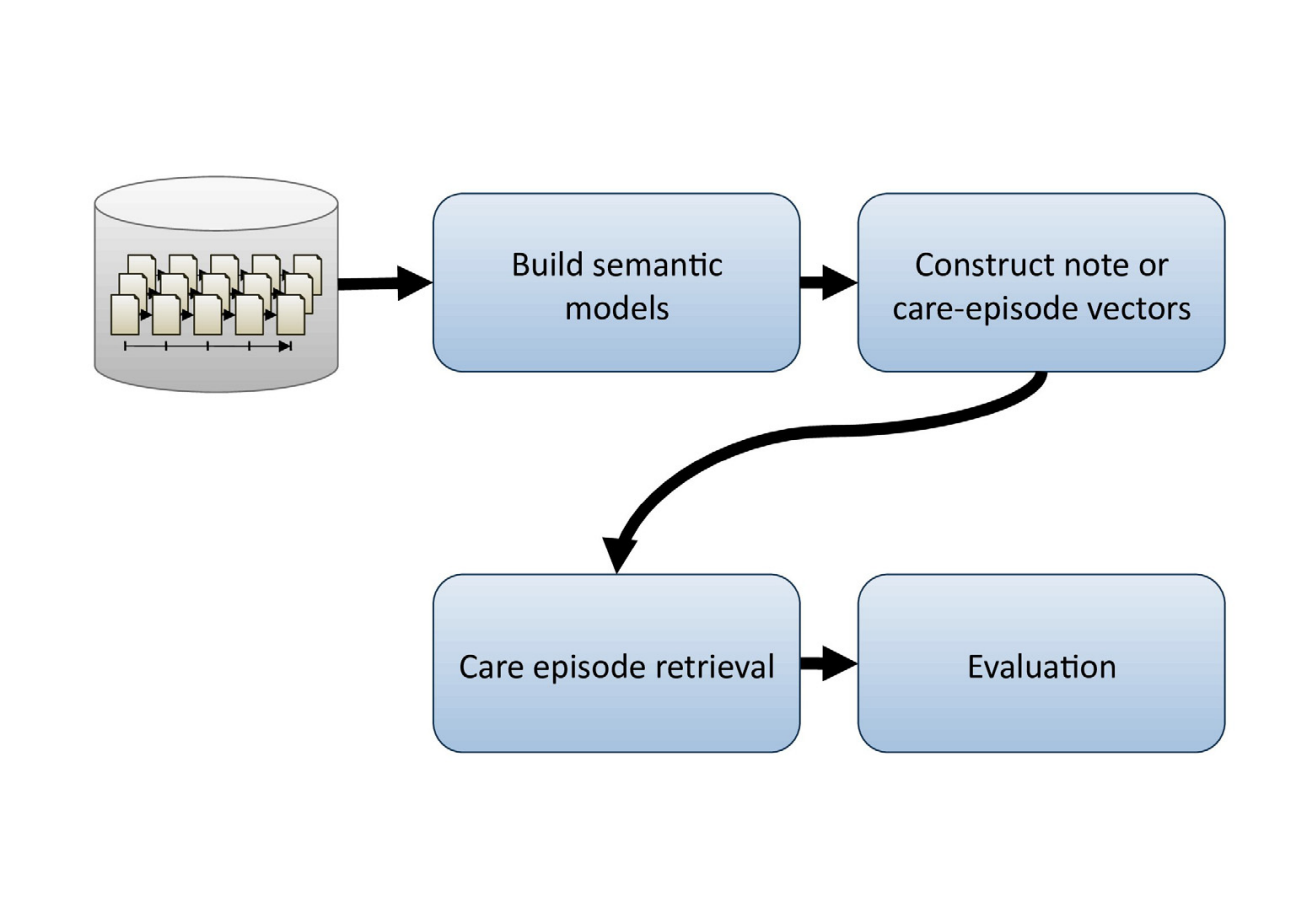
**Experimental setup overview**. Figure shows an overview of the various steps in our experimental setup.

The task addressed in this study is retrieval of care episodes that are similar to each other in terms of their clinical free text. The purpose is to explore how a set of different distributional models perform in care episode retrieval, and also to determine how care episode similarity is best calculated. In contrast to the normal IR setting, where the search query is derived from a text stating the user's information need, here the query is based on another care episode, which we refer to as the *query episode*. As the query episode may document ongoing treatment, and thus lack a discharge summary and ICD-10 code, neither of these information sources can be relied upon for constructing the query. The task is therefore to retrieve the most similar care episodes using only the information contained in the free text of the clinical notes in the query episode. An overview showing the steps in our experimental setup is illustrated in Figure 2.

Evaluation of retrieval results generally requires an assessment of their relevancy to the query. To perform automatic evaluation, a *gold standard *is needed, which specifies the relevant documents from the collection for each query. It is common to produce such a gold standard through (semi-) manual work, relying on multiple human experts to select or rank documents according to their relevancy to a given query. Hence, obtaining such judgements is typically costly and time-consuming. Moreover, for the care episode retrieval task, the manual work would require experts in the clinical domain.

In relation to this study, with the help of an expert in the clinical domain, we tried to assess the feasibility of creating such a gold standard for the care episode retrieval task. What we found was that assessing whether or not two care episodes are similar, strictly based on the information in both texts, was a difficult task with a lot of room for (individual) interpretation, especially for the top-ranked care episodes. By looking at the top-10 care episodes retrieved by the various semantic models and Lucene for a particular query episode, we found almost all of them had many overlapping clinical features with the query episode, even if they did not share the same primary ICD-10 code. In many cases they shared ICD-10 codes, but not necessarily the primary ones. Also, even though many patients were hospitalized due to similar reasons and/or backgrounds, this did not necessarily mean that they were treated in response to the exact same diagnosis, given the same treatments, or received those treatments in the same order. We estimate the two most time-consuming sub-tasks to be (1) creating explicit and unambiguous guidelines for the human evaluators, possibly unique ones for each query episode; (2) performing the evaluation for the required number of care episodes (average being 357 care episodes for each of the 40 queries when looking at the top 100 retrieved care episodes per query for each model/system). In addition, it is important to have enough human evaluators evaluating the same data to be able to verify that inter-annotator agreement is of a sufficiently high level. We concluded that the effort required for creating such a gold standard was simply too much for the resources we had access to.

As we did not have access to the required resources for creating the evaluation set manually, we opted for an alternative approach. Two different evaluation strategies were used in an attempt to approximate human relevance judgements. The first evaluation method is based on the assumption that a retrieved episode is relevant if its ICD-10 code is identical to that of the query episode. The second method assumes that a retrieved episode is relevant if its discharge summary is semantically similar to that of the query episode. In this setting, crucially, discharge summaries or ICD-10 codes are not used in either query construction or episode retrieval. Both evaluation methods are further detailed in the sections 'Experiment 1: ICD-10 code identity' and 'Experiment 2: Discharge summary overlap' respectively.

## Data

The data set used in this study consists of the electronic health records from patients with any type of heart related problem that were admitted to one particular university hospital in Finland between the years 2005-2009. Of these, the clinical notes written by physicians are used (i.e. we did not use the corresponding nursing notes). An assent for the research was obtained from the Ethics Committee of the Hospital District (17.2.2009 §67) and permission to conduct the research was obtained from the Medical Director of the Hospital District (2/2009). The total data set consists of 66884 care episodes, which amounts to 398040 notes and 64 million words in total. Words here refer to terms identified through the lemmatization, except terms being pure numbers. This full set was used for training of the semantic models. To reduce the computational demands of experimentation, a subset was used for evaluation purposes, comprising 26530 care episodes, amounting to 155562 notes and 25.7 million words in total.

Notes are mostly unstructured, consisting of Finnish clinical free text.

The care episodes have been manually labeled according to ICD-10. Codes are normally applied at the end of the patients' hospital stay, or even after the patient has been discharged from the hospital. Care episodes have commonly one primary ICD-10 code attached and optionally a number of additionally secondary codes. As extraction of the potential secondary ICD-10 codes is non-trivial, we have in this study only used the primary ICD-10 codes - used for training two of the semantic models and for evaluation purposes in Experiment 1.

ICD-10 codes have an internal structure that reflects the classification system ranging from broad categories down to fine-grained subjects. For example, the first character (J) of the code J21.1 signals that it belongs to the broad category *Diseases of the respiratory system*. The next two digits (21) classify the subject as belonging to the subcategory *Acute bronchiolitis*. Finally, the last digit after the dot (1) means that it belongs to the sub-subclass *Acute bronchiolitis due to human metapneumovirus*. There are 356 unique "primary" ICD-10 codes in the evaluation data set.

## Methods

### Semantic models

A crucial part in retrieving similar care episodes is having a good similarity measure. Here similarity between care episodes is measured as the semantic similarity between the words they contain (see section 'Compute care episode similarity'). Semantic similarity between words is in turn measured through the use of distributional semantic models. In this way, no explicit query expansion step is performed, but potentially indirect word matches are found through the various models. Several model variants are tested, utilizing different techniques and parameters for building them. The models trained and tested in this paper are: (1) classic random indexing with a sliding window using term index vectors and term context vectors (RI-Word); (2) random indexing with index vectors for clinical notes (RI-Note); (3) random indexing with index vectors for ICD-10 codes (RI-ICD); (4) a version of random indexing where only the term index vectors are used (RI-Index); (5) a semantic neural network model, using *word2vec *(W2V) to build word context vectors (W2V); and (6) a W2V version of the RI-ICD method, using a modified version of W2V for training (W2V-ICD).

#### RI-Word

**Figure 3:**
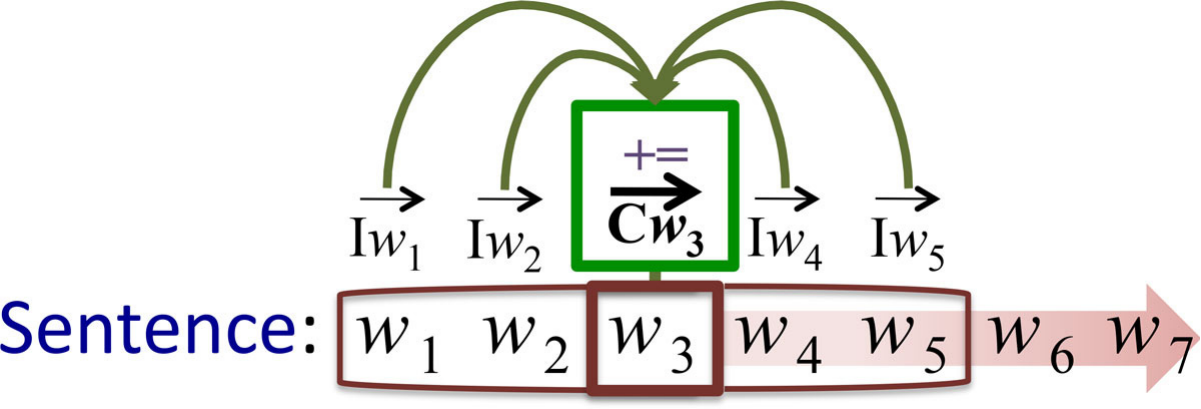
**Training the RI-Word model**. A sliding window with a size of five words is moved over the text, word by word. The context vector $\vec{\text{C}w_{3}}$ for the word in the center of the window *w*_3 _is updated by adding the index vectors of the other words within the window, i.e. $\vec{\text{I}w_{1}}$, $\vec{\text{I}w_{2}}$, $\vec{\text{I}w_{4}}$ and $\vec{\text{I}w_{5}}$. As a result, context vector $\vec{\text{C}w_{3}}$ records the fact that *w*_3 _co-occurs with word *w*_1_, *w*_2_, *w*_4 _and *w*_5_. The training process continues with moving the sliding window one position to the right and repeating the addition operation for context vector $\vec{\text{C}w_{4}}$, and so on until the end of the training text is reached.

Random indexing (RI) [48] is a method for building a (pre) compressed vector space model with a fixed dimensionality, done in an incremental fashion. RI involves the following two steps: First, instead of allocating one dimension in the multidimensional vector space to a single word, each word is assigned an "index vector" as its unique signature in the vector space. Index vectors are generated vectors consisting of mostly zeros together with a randomly distributed set of several 1's and -1's, uniquely distributed for each unique word; the second step is to induce "context vectors" for each word. A context vector represents the *contextual meaning *of a word. This is done using a sliding window of a fixed size to traverse a training corpus, inducing context vectors for the center/target word of the sliding window by summing the index vectors of the neighbouring words in the window. An example illustrating how RI-Word works is shown in Figure 3.

As the dimensionality of the index vectors is fixed, the dimensionality of the vector space will not grow beyond the size *W × Dim*, where *W *is the number of unique words in the vocabulary, and *Dim *being the pre-selected dimensionality to use for the index vectors. As a result, RI models are significantly smaller than plain vector space models, making them a lot less computationally expensive. Additionally, the method is fully incremental (additional training data can be added at any given time without having to retrain the existing model), easy to parallelize, and scalable, meaning that it is fast and can be trained on large amounts of text in an on-line fashion.

#### RI-Note

**Figure 4:**
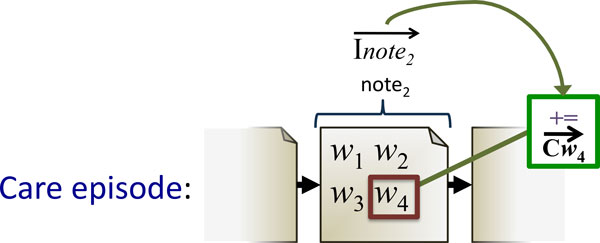
**Training the RI-Note model**. Word *w*_4_'s context vector, $\vec{\text{C}w_{4}}$, is updated by adding the index vector $\vec{\text{I}note_{2}}$ of the note it is part of. The same update is applied to all other words in the note. As a result, context vectors for words co-occurring in the same note become more similar.

Contrary to sliding window approach used in RI-Word, a RI model built with *note index vectors *first assigns unique index vectors to every clinical note in the training corpus. In the training phase, each word in a note gets the corresponding note index vector added to its context vector. See Figure 4 for an illustration of how RI-Note works.

#### RI-ICD

**Figure 5:**
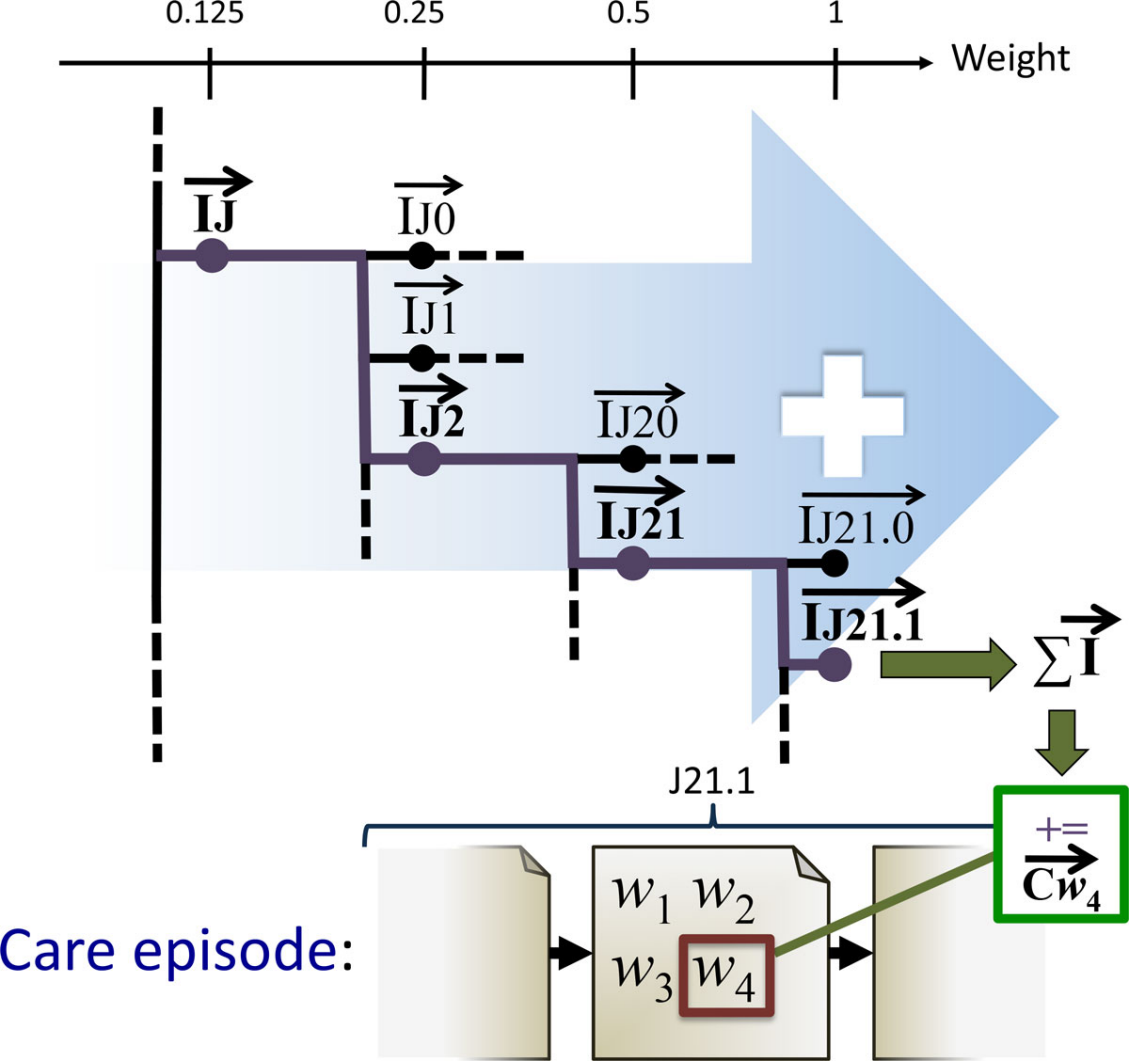
**Training the RI-ICD model**. Word *w*_4 _occurs in a note that is part of a care episode labeled with the ICD-10 code J21.1. Its context vector $\vec{\text{C}w_{4}}$ is therefore updated by adding the index vector for the code J21.1. This context vector is constructed from the weighted sum of index vectors of its parts: $\sum \vec{\text{I}}=\left(0.125\times \vec{{\text{I}}_{\text{J}}}\right)+\left(0.25\times \vec{{\text{I}}_{\text{J}2}}\right)+\left(0.5\times \vec{{\text{I}}_{\text{J}21}}\right)+\left(1.0\times \vec{{\text{I}}_{\text{J}21.1}}\right)$. As a result, *w*_4_'s context vector becomes more strongly associated with the code J21.1 and - to a lesser degree - with all superclasses of J21.1 in the ICD-10 taxonomy. The same update is applied to the context vectors of all other words in care episodes labeled as J21.1.

Based on the principle of RI with note index vectors, we here explore a novel method for constructing a vector space model by exploiting the ICD-10 code classification done by clinicians. Instead of using note index vectors, we now use *ICD-code index vectors*. First, a unique index vector is assigned to each chapter and sub-chapter in the ICD-10 taxonomy. This means assigning a unique index vector to each "node" in the ICD-10 taxonomy, as illustrated in Figure 5. For each clinical note in the training corpus, the index vector of the their primary ICD-10 code is added to all words within it. In addition, all the index vectors for the ICD-codes higher in the taxonomy are added, each weighted according to their position in the hierarchy. A weight of 1 is given to the full code, while the weight is halved for each step upwards in the hierarchy. The motivation for the latter is to capture a certain degree of similarity between codes that share an initial path in the taxonomy. As a result, this similarity gets encoded in the resulting model. As an example, illustrated in Figure 5: for a clinical note labelled with the code J21.1, we add the following index vectors to the context vectors of all its constituting words: $\vec{{\text{I}}_{\text{J}}}\times 0.125,\vec{{\text{I}}_{\text{J}2}}\times 0.25,\vec{{\text{I}}_{\text{J}21}}\times 0.5$ and $\vec{{\text{I}}_{\text{J}21.1}}\times 1.0$. The underlying hypothesis for building a model in this way is that it may capture relations between words in a way that better reflects the clinical domain, compared with the other domain-independent methods for modelling.

#### RI-Index

As an alternative to using context vectors for words, we simply only use their index vectors in place of context vectors, therefore not modelling their "contextual meaning". When constructing note or care episode vectors directly from word index vectors (see the 'Compute care episode similarity' section), the resulting vectors represent a compressed version of a TF*IDF matrix, which again is similar to Lucene.

#### W2V

Recently, a novel method for inducing vector space models was introduced by Mikolov et al. [42], stemming from the research in deep learning and neural network language models. While the overall objective of learning a continuous vector space representation for each word based on its textual context remains, the underlying algorithms are substantially different from traditional methods such as LSA and RI. The model is based on a somewhat simplified neural network with as many input nodes as there are vocabulary items, a hidden linear projection layer with as many nodes as is the desired dimensionality of the vector space, and finally a hierarchical soft-max output layer. Every node in the hidden projection layer calculates a linear combination of the values of the input layer nodes (0 or 1), as its own value. The nodes of the output layer, in turn, calculate a linear combination of the hidden layer node outputs, which is subsequently passed through a specific non-linear function. The network is trained one input-output example pair at a time, and for each pair the difference between the expected and the actual output of the network is calculated. The linear combination weights in the network are subsequently adjusted to decrease the error using the *back-propagation *procedure. This procedure is repeated for all training data pairs, often in several passes over the entire training dataset, until the network converges and the error does not decrease any further. The application of neural networks specifically in word prediction tasks is presented, for example, by Bengio et al. [53].

**Figure 6:**
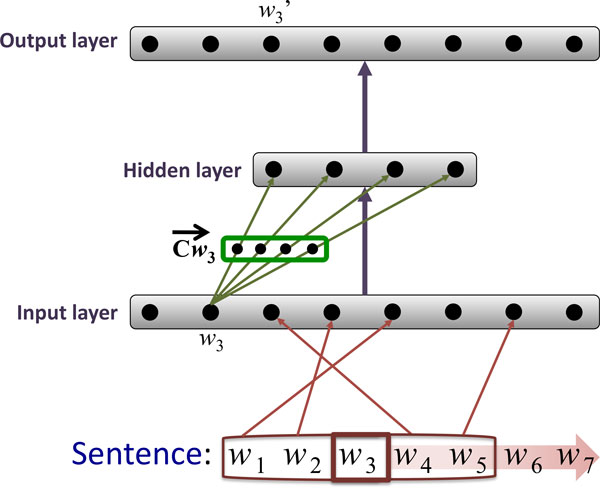
**Training the W2V BoW model**. A sliding window with the size of five words is moved over the text, word by word. The input layer nodes of the network corresponding to the words in the context window of the word *w*_3 _are activated. The error in the output layer prediction and the expected prediction for the focus word *w*_3 _is back-propagated through the network. When the training is completed, the context vector $\vec{\text{C}w_{3}}$ constitutes the set of weights connecting the input layer node for *w*_3 _and the hidden layer.

The main distinguishing features specific to the W2V model are the linear (as opposed to the traditionally non-linear) hidden layer, and the usage of the efficient hierarchical soft-max output layer, which allows for a substantial decrease in the number of output nodes that need to be considered for the back-propagation. Combined, these two techniques allow the network to be efficiently trained on billions of tokens worth of input text. There are two distinct regimes in which the network is trained, or in other words, two ways to define the task on which the network is trained. In the *skip-gram *architecture, the network is trained given a focus word to predict a nearby word. I.e. a sliding window of typically *±*10 tokens wide is slid across the text with the focus word at its center and each word within the window is in turn considered a prediction target. The focus word - context word pairs then constitute the word pairs used to train the network. The single input node corresponding to the focus word is activated while setting all other input layer nodes to zero (also referred to as *one hot *representation), and the error in the output layer prediction of the context word is back-propagated through the network. It is important to note that the output layer predictions are only necessary to train the network and we are not interested in them otherwise. To understand on intuitive level why the network learns efficient representations, consider the two-step process of the prediction: first, the input layer is used to activate the hidden, representation layer and second, the hidden layer is used to activate the output layer and predict the context word. To maximize the performance on this task, the network is thus forced to assign similar hidden layer representations to words which tend to have similar contexts. Since these representations form the W2V model, distributionally similar words are given similar vector representations. An alternative training regime is the *BoW *(bag of words) architecture. In this architecture, all words from the context are used at once to activate the respective nodes in the input layer, and the focus word is the prediction target. In a sense, it is the reverse of the skip-gram architecture. The main advantage of the BoW regime is its speed, because only a single update of the network is necessary per each context, unlike in the skip-gram architecture, where as many updates are performed as there are words in the context. Regardless of the training regime, the vector space representation of a word is the weight vector from its corresponding input node to the hidden layer. As mentioned previously, the hidden-to-output layer weights are discarded after training. See Figure 6 for an example illustrating how model training with W2V is carried out.

One of the main practical advantages of the W2V method lies in its scalability, allowing the training on billions of words of input text in the matter of several hours, setting it apart from the majority of other methods of distributional semantics. Additionally, the W2V method has been shown to produce representations that preserves important linguistic regularities [54]; as elaborated by Levy and Goldberg [55].

#### W2V-ICD

**Figure 7:**
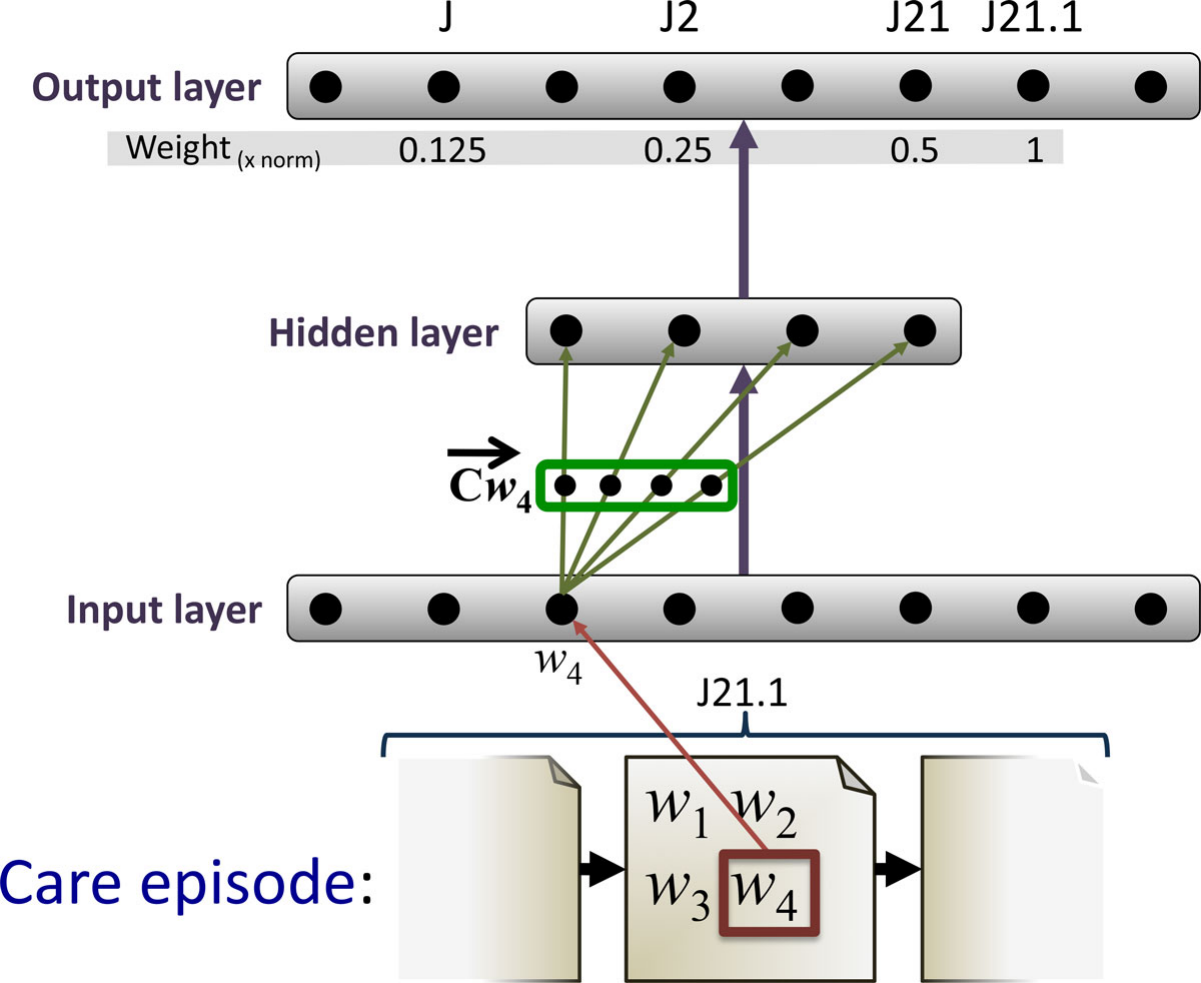
**Training the W2V-ICD model**. Word *w*_4 _occurs in a note that is part of a care episode labeled with the ICD-10 code J21.1. The input node corresponding to *w*_4 _is activated and the error between the output layer prediction and the expected prediction for J21.1 is back-propagated through the network. Same procedure is repeated for J21, with the update scaled by 0.5, and J2 scaled by 0.25, and finally J, scaled by 0.125. When the training is completed, the context vector $\vec{\text{C}w_{4}}$ is formed by the weights connecting the input node corresponding to *w*_4 _and the hidden layer of the network.

As will be shown, the RI-ICD method offers a notable advantage over the standard RI in the care episode retrieval task. We therefore introduce a novel variant of the W2V algorithm which implements the same insights as the RI-ICD method. As the starting point serves the fact that only the input-to-hidden layer weights define the final vector space representation. Therefore, as long as we preserve the input and hidden layers as in the original W2V architecture, i.e. a single input node for every word and a hidden layer with as many nodes as is the dimensionality of the representation, we are free to modify the prediction task of the network. In this case, we will use the ICD-10 codes for the corresponding clinical notes as the prediction target, training the network to predict the ICD-10 code of the note given a word from it. Following a similar intuition as for the skip-gram architecture, in order to maximize the performance on the task, the network will assign similar representation to words which occur in notes with the same ICD-10 codes. This objective clearly mirrors the motivation for the RI-ICD method. As in RI-ICD, we make use of the hierarchical structure of the ICD-10 codes, as illustrated in Figure 5, whereby not only the full ICD-10 code, but also its structural parts constitute training targets for the network. For each note, the network is thus trained on all pairs of a word from the note on the input layer, and a structural segment of the ICD-10 code as the prediction target. We use the ICD-10 code segments and their frequencies to define a vocabulary, whereupon we induce the hierarchical soft-max layer in exactly the same manner as in the standard W2V algorithm. We implement the exact same weighting as in the RI-ICD method, giving ICD-10 code segments a weight which decreases as the generality of the segment increases. We then use these weights to scale the update gradient propagated through the network. See Figure 7 for an example how this training is done.

### Compute care episode similarity

After having computed a semantic model, or six in this case, together with the baselines, the next step is to calculate care episode similarities for the retrieval task. Multiple ways of calculating care episode similarities exist.

We explore two overall approaches: One where each care episode is viewed as a single document, with all corresponding notes concatenated (SingleSim); Another where each care episode is viewed as a set of individual notes. For the latter, care episode similarity between two care episodes is calculated from pairwise note similarities and aggregating into a single similarity score. This again can be done in multiple ways. Three approaches are explored here (AvgSim, HASim and NWSim).

#### SingleSim: Single care episode vectors

Here we compute a separate vector for each care episode by summing the word vectors for all words in the care episode. The resulting vector is divided by the total number of words in the episode to normalize for differences in length between care episodes. Similarity between care episodes is then determined by computing the cosine similarity between their vectors.

#### AvgSim: Average note vector similarity

Each individual note within a care episode gets its own note (document) vector by summing the word vectors for all words in the note. In order to compute the similarity between two episodes, we take the average over the exhaustive pairwise similarities between their notes. That is, for every note in the first care episode, we compute its similarity to every note in the second care episode, and then take the average over all these pairwise similarities. Similarity between notes is again measured by the cosine similarity between their vectors.

#### HASim: Hungarian Algorithm for pairing of note vectors

**Figure 8:**
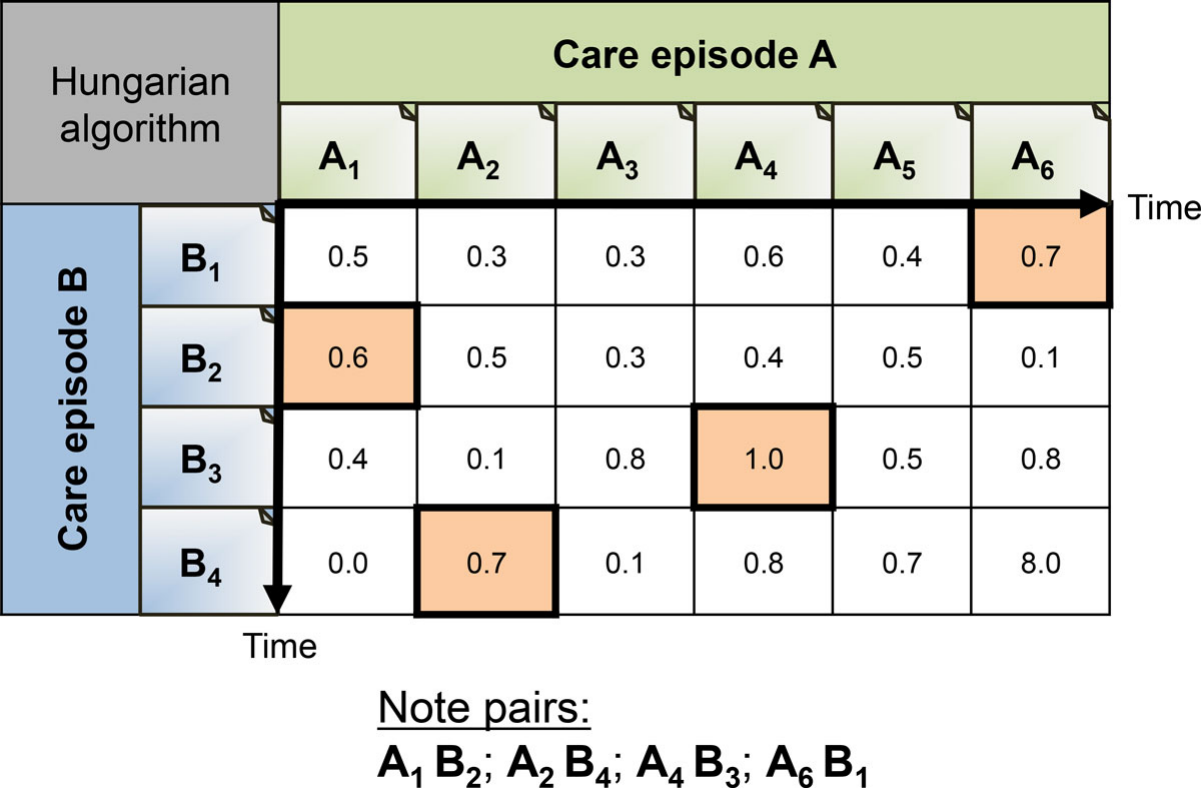
**Hungarian algorithm for note pairing**. Figure showing an example of how the Hungarian algorithm would find the optimal clinical note pairs for care episode A and B.

For two care episodes, a note-to-note similarity matrix is calculated, populated with pairwise note vector similarities. By applying the *Hungarian Algorithm *[56], we compute the optimal pairing of each note in one episode to exactly one other note in the other episode, maximizing the sum of similarities. The final similarity between two care episodes is this sum of their paired notes similarities, multiplied by two, and divided by the total number of notes in the two care episodes (Equation 1). See Figure 8 for an example showing how the notes are paired using the Hungarian Algorithm.

$$Sim\left(A,B\right)=\frac{2\times \sum CosSim\left(\vec{A_{i}},\;\vec{B_{j}}\right)}{A.noteCount+B.noteCount}$$

#### NWSim: Needleman-Wunsch algorithm for sequence alignment of note vectors

**Figure 9:**
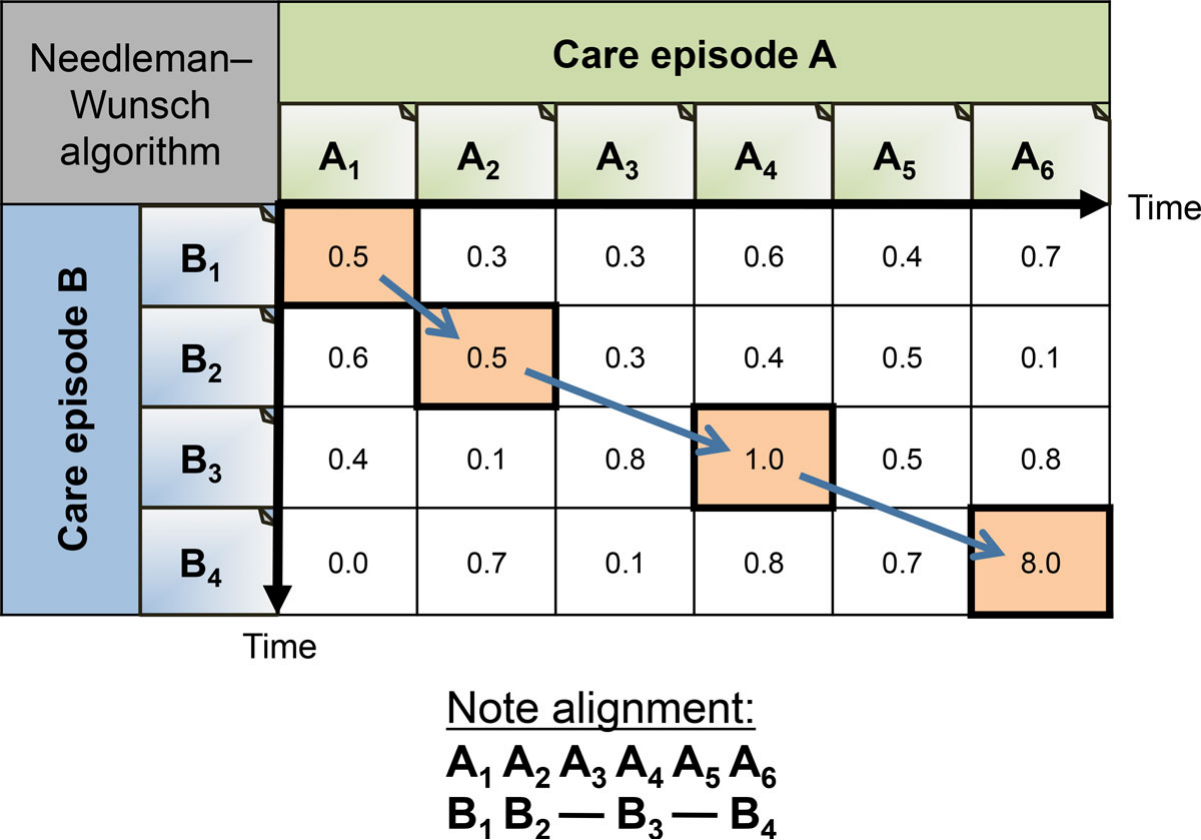
**Needleman-Wunsch algorithm for note alignment**. Figure showing an example of how the Needleman-Wunsch algorithm would align clinical note pairs for care episode A and B.

Here we utilize a sequence alignment algorithm called *Needleman-Wunsch *[57] to align two episodes by their most similar notes. A note in one care episode can be aligned with zero or one notes in the other care episode. See Figure 9 for an example showing how the notes are aligned using the Needleman-Wunsch algorithm. The difference with the Hungarian Algorithm is that the temporal order of the notes is preserved. In other words, crossing alignment are not allowed. This reflects the intuition that care episodes sharing treatments in the same order are more likely to be similar than episodes with the same treatments in a different temporal order. We found that using the overall score produced by the Needleman-Wunsch algorithm for care episode similarity did not give any good results at this task. Instead, similarity between two care episodes is calculated from pairwise note vector similarities for the aligned notes. Final care episode similarity scores are obtained by using Equation 1.

### Word vector weighting

The word vectors used in calculating care episode similarities, as described in section 'Compute care episode similarity', are all normalized and weighted before they are used. Common to all is that they are first normalized and multiplied by their Inverse Document Frequency (IDF) weight [58]. Such weighting is done in an attempt to weight words by their overall relevancy compared to the other words on corpus level. It essentially gives more weight to words occurring in few documents (notes in our case) while giving less weight to those occurring in many documents. We refer to this weighting method as IDFWeight.

As a part of the experiment reported here, we aim to improve upon the domain-independent IDF weighting. For this, we boost the weight of words with clinical relevancy. This is accomplished by doubling the IDF weight of words occurring in a Finish health metathesaurus [59], which contains terms extracted from: vocabularies and classifications from FinMeSH; ICD-10; ICPC-2 (International Classification of Primary Care); the ATC-classification (generic drug names by WHO); the NOMESCO classification for surgical procedures; the Finnish vocabulary on nursing. This weighting method will be referred to as IDF*MetathesaurusWeight. Each of the approaches to calculating care episode similarity, with the models described in section 'Semantic models', are tested both with and without such metathesaurus-based re-weighting of word vectors.

### Baselines

Two baselines were used in this study. The first one is random retrieval of care episodes, which can be expected to give very low scores and serves merely as a sanity check. The second one is Apache Lucene [29], a state-of-the-art search engine based on look-up of similar documents through a reverse index and relevance ranking based on a TF*IDF-weighted vector space model. Care episodes and underlying notes were indexed using Lucene. Similar to the other models/methods, all of the free text in the query episode, excluding the discharge summary and any sentence mentioning ICD-10 codes, served as the query string provided to Lucene. Being a state-of-the-art IR system, the scores achieved by Lucene in these experiments should indicate the difficulty of the task.

## Results

### Experiment 1: ICD-10 code identity

As explained in the 'Task' section, we lack a gold standard data set for care episode retrieval, consisting of relevant documents per query according to judgements by human experts. Therefore the relevance of retrieved episodes is estimated using a proxy. In this experimental setup, evaluation is based on the assumption that a retrieved episode is relevant if its ICD-10 code is identical to that of the query episode. It should be stressed that ICD-10 codes, i.e. possible free-text sentences that mention an ICD-10 code, are not included in the queries when conducting the experiment.

In the experiment we strove to have a setup that was as equal as possible for all models and systems, both in terms of text pre-processing and in terms of the target model dimensionality when inducing the vector space models. The clinical notes are split into sentences, tokenized, and lemmatized using a Constraint-Grammar based morphological analyzer and tagger extended with clinical vocabulary [60]. After stop words were removed [61], the total training corpus contained 39 million words (minus the query episodes), while the evaluation subset contained 18.5 million words. The vocabulary consisted of 0.6 million unique words.

In total, 40 care episodes were randomly selected to serve as the query episodes during testing, with the requirement that each had different ICD-10 codes and consisted of a minimum of six clinical notes. The average number of words per query episode is 796. The number of correct episodes per query episode varies between 9 and 1654. The total is 18440 episodes with an average length of 461 words per episode. When conducting the experiment all care episodes were retrieved for each of the 40 query episodes.

The RI- and W2V-based models have all a predefined dimensionality of 800. For the RI-based models, 4 non-zeros were used in the index vectors. For the RI-Word model, a narrow context window was employed (5 left + 5 right), weighting index vectors according to their distance to the target word $\left(weight_{i}=2^{1-dist_{it}}\right)$. In addition, the index vectors were shifted once left or right depending on what side of the target word they were located, similar to *direction vectors *as described in Sahlgren et al. [62]. These parameters for RI were chosen based on previous work on semantic textual similarity [63]. Also a much larger window of 20+20 was tested, but without noteworthy improvements.

The W2V-based models are trained using the BoW architecture and otherwise default parameters. The W2V-ICD model is trained with 10 iterations with a progressively decreasing learning rate, starting from 0.04. The utilized software was: Apache Lucene (version 4.2.0) [29]; The word2vec tool [64], for training the W2V model; A modified W2V implementation from the gensim library [65], for training of the W2V-ICD-based models; JavaSDM package [66], which served as the basis for the RI-based methods. Evaluation scores were calculated using the *TREC eval *tool [67].

As we have two different word vector weighting methods, and four different ways to calculate care episode similarities, a total of eight test runs was conducted:

• IDFWeight & SingleSim (Table 1).

**Table 1:** Experiment 1: Results from the IDFWeight & SingleSim setup.

IR model	MAP	P@10	Rprec
**Lucene**	0.1210	0.2800	0.1527
**RI-Word**	0.0915	0.2475	0.1300
**RI-Note**	0.1035	0.2850	0.1356
**RI-ICD**	0.2478	0.4250	0.2601
**RI-Index**	0.1171	0.3075	0.1555
**W2V**	0.1557	0.3000	0.1892
**W2V-ICD**	0.2666	0.3975	0.2874
**Random**	0.0178	0.0175	0.0172

• IDFWeight & AvgSim (Table 2).

**Table 2:** Experiment 1: Results from the IDFWeight & AvgSim setup.

IR model	MAP	P@10	Rprec
**Lucene**	0.0915	0.1564	0.0963
**RI-Word**	0.0317	0.0667	0.0465
**RI-Note**	0.0530	0.1308	0.0701
**RI-ICD**	0.1481	0.2256	0.1645
**RI-Index**	0.0599	0.1026	0.0654
**W2V**	0.1200	0.2128	0.1510
**W2V-ICD**	0.2357	0.3462	0.2499
**Random**	0.0178	0.0175	0.0172

• IDFWeight & HASim (Table 3).

**Table 3:** Experiment 1: Results from the IDFWeight & HASim setup.

IR model	MAP	P@10	Rprec
**Lucene**	0.1045	0.2385	0.1230
**RI-Word**	0.0319	0.1154	0.0456
**RI-Note**	0.0425	0.1487	0.0639
**RI-ICD**	0.0464	0.2333	0.0640
**RI-Index**	0.0840	0.2231	0.1112
**W2V**	0.0791	0.2513	0.1124
**W2V-ICD**	0.0917	0.2359	0.1311
**Random**	0.0178	0.0175	0.0172

• IDFWeight & NWSim (Table 4).

**Table 4:** Experiment 1: Results from the IDFWeight & NWSim setup.

IR model	MAP	P@10	Rprec
**Lucene**	0.0812	0.2282	0.0938
**RI-Word**	0.0288	0.0795	0.0384
**RI-Note**	0.0358	0.0486	0.1000
**RI-ICD**	0.0407	0.1821	0.0560
**RI-Index**	0.0552	0.1923	0.0742
**W2V**	0.0647	0.1949	0.0954
**W2V-ICD**	0.0938	0.2410	0.1264
**Random**	0.0178	0.0175	0.0172

• IDF*MetathesaurusWeight & SingleSim (Table 5).

**Table 5:** Experiment 1: Results from the IDF*MetathesaurusWeight & SingleSim setup.

IR model	MAP	P@10	Rprec
**Lucene**	0.1210	0.2800	0.1527
**RI-Word**	0.0958	0.2600	0.1355
**RI-Note**	0.1161	0.2975	0.1501
**RI-ICD**	0.2372	0.4200	0.2541
**RI-Index**	0.1387	0.3100	0.1775
**W2V**	0.1619	0.3125	0.1968
**W2V-ICD**	0.2580	0.3850	0.2793
**Random**	0.0178	0.0175	0.0172

• IDF*MetathesaurusWeight & AvgSim (Table 6).

**Table 6:** Experiment 1: Results from the IDF*MetathesaurusWeight & AvgSim setup.

IR model	MAP	P@10	Rprec
**Lucene**	0.0915	0.1564	0.0963
**RI-Word**	0.0313	0.0641	0.0455
**RI-Note**	0.0559	0.1385	0.0741
**RI-ICD**	0.1453	0.2462	0.1632
**RI-Index**	0.0680	0.1000	0.0732
**W2V**	0.1280	0.2333	0.1592
**W2V-ICD**	0.2280	0.3410	0.2454
**Random**	0.0178	0.0175	0.0172

• IDF*MetathesaurusWeight & HASim (Table 7).

**Table 7:** Experiment 1: Results from the IDF*MetathesaurusWeight & HASim setup.

IR model	MAP	P@10	Rprec
**Lucene**	0.1045	0.2385	0.1230
**RI-Word**	0.0318	0.1128	0.0451
**RI-Note**	0.0430	0.1538	0.0631
**RI-ICD**	0.0452	0.2256	0.0627
**RI-Index**	0.0930	0.2385	0.1225
**W2V**	0.0814	0.2308	0.1176
**W2V-ICD**	0.0874	0.2359	0.1257
**Random**	0.0178	0.0175	0.0172

• IDF*MetathesaurusWeight & NWSim (Table 8).

**Table 8:** Experiment 1: Results from the IDF*MetathesaurusWeight & NWSim setup.

IR model	MAP	P@10	Rprec
**Lucene**	0.0812	0.2282	0.0938
**RI-Word**	0.0288	0.0872	0.0379
**RI-Note**	0.0354	0.1179	0.0500
**RI-ICD**	0.0393	0.1821	0.0537
**RI-Index**	0.0601	0.2231	0.0810
**W2V**	0.0663	0.2051	0.0972
**W2V-ICD**	0.0890	0.2333	0.1196
**Random**	0.0178	0.0175	0.0172

Performance on care episode retrieval was assessed using three different evaluation measures:

• *Precision at 10 *(P@10) denotes the precision among the top-10 results, in other words, the proportion of episodes with the same ICD-10 code as the query episode among the first 10 retrieved episodes. This probably best reflects the clinical usage scenario where a user is only prepared to check the highest ranked results, but is not willing to go through all results. P@10 scores reported are averages over 40 queries.

• R-precision (Rprec) is defined as the precision at the R-th position in the results, where R is the number of correct entries in the gold standard. This indicates the proportion of the top-R retrieved episodes with the same ICD-10 code as the query episode, where R is the number of episodes with the same ICD-10 code in the whole collection. Our Rprec scores are averages over 40 queries.

• Mean average precision (MAP) is defined as the mean of the average precision over all (40) queries. For each query, the average is the precision value obtained for the top k documents, each time a relevant doc is retrieved. This is probably the most commonly used evaluation measure in IR. Moreover, it is very sensitive to ranking, so systems that rank the most similar episodes first receive higher MAP scores.

For the models using normal IDF weighting of word vectors (IDFWeight) the MAP, P@10 and Rprec results from each model, baselines, and the different ways to calculate care episode similarities, are shown in Tables 1, 2, 3, and 4. More precisely, results using IDFWeight and SingleSim are shown in Table 1. Table 2 shows the results from IDFWeight and AvgSim. Table 3 shows the results from IDFWeight and HASim. Table 4 shows the results from IDFWeight and NWSim. Best overall results among these are achieved with the setup SingleSim. Here, model W2V-ICD achieves highest MAP and Rprec scores, closely followed by RI-ICD. RI- ICD achieves the best P@10 scores. For the other setups, where each care episode is viewed as a collection of notes, shown in Tables 2, 3 and 4, the AvgSim approach to calculating care episode similarities achieves highest scores for most models. The exceptions are Lucene and RI-Index (and P@10 scores for RI-Word), which achieve noteworthy better scores with the HASim approach. No models achieve best scores with the NWSim approach. On average, W2V, W2V-ICD and RI-ICD outperforms Lucene. The other models either achieve scores that are comparable to Lucene, or lower. The latter is especially the case for the AvgSim, HASim and NWSim. Lucene and RI-Index seem to somewhat follow each other in terms of performance, which is as expected, given the similarities in how they are trained.

For the models using IDF weighting and double weight to words matching those in a health metathesaurus (IDF*MetathesaurusWeight), results are shown in Tables 5, 6, 7, and 8. The same trends are seen here with regards to scoring, where all models achieve best scores with SingleSim. No performance is gained in viewing each care episode as a collection of multiple individual notes.

When comparing the differences between IDFWeight (Tables 1, 2, 3, and 4) with IDF*MetathesaurusWeight (Tables 5, 6, 7, and 8), most setups and models achieve increased scores with IDF*MetathesaurusWeight. This is however not the case for the two models relying on ICD-10 codes for training, namely RI-ICD and W2V- ICD.

### Experiment 2: Discharge summary overlap

This experiment uses a different evaluation method in which the semantic similarity between discharge summaries is used as a proxy for relevance. It assumes that a retrieved episode is relevant if its discharge summary is semantically similar to that of the query episode. It should be emphasized that discharge summaries are not used in either query construction or episode retrieval. Using the discharge summaries of the query episodes, the top 100 care episodes with the most similar discharge summary were selected. This procedure was repeated for each model - i.e. the six different semantic models and Lucene - resulting in seven different test sets, each consisting of 40 query episodes with their corresponding 100 most similar collection episodes. The top 100 was used rather than a threshold on the similarity score, because otherwise seven different thresholds would have to be chosen.

**Figure 10:**
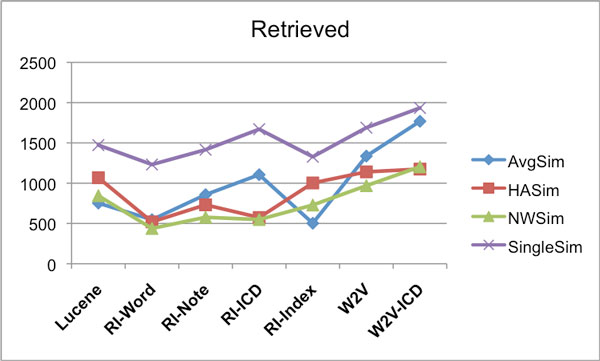
**IDFWeight-Eval - IDFWeight-Results - Retrieved counts**. Average number of correctly retrieved care episodes (max 4000) for different similarity measures using the various IR models. For creating the evaluation set the IDFWeight weighting was used, and also the retrieval was done using the IDFWeight weighting.

**Figure 11:**
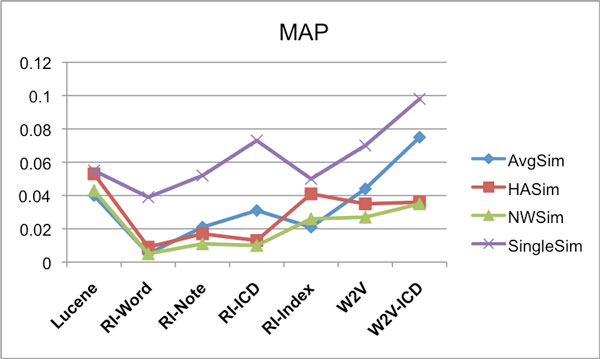
**IDFWeight-Eval - IDFWeight-Results - MAP**. Average MAP scores for different similarity measures using the various IR models. For creating the evaluation set the IDFWeight weighting was used, and also the retrieval was done using the IDFWeight weighting.

**Figure 12:**
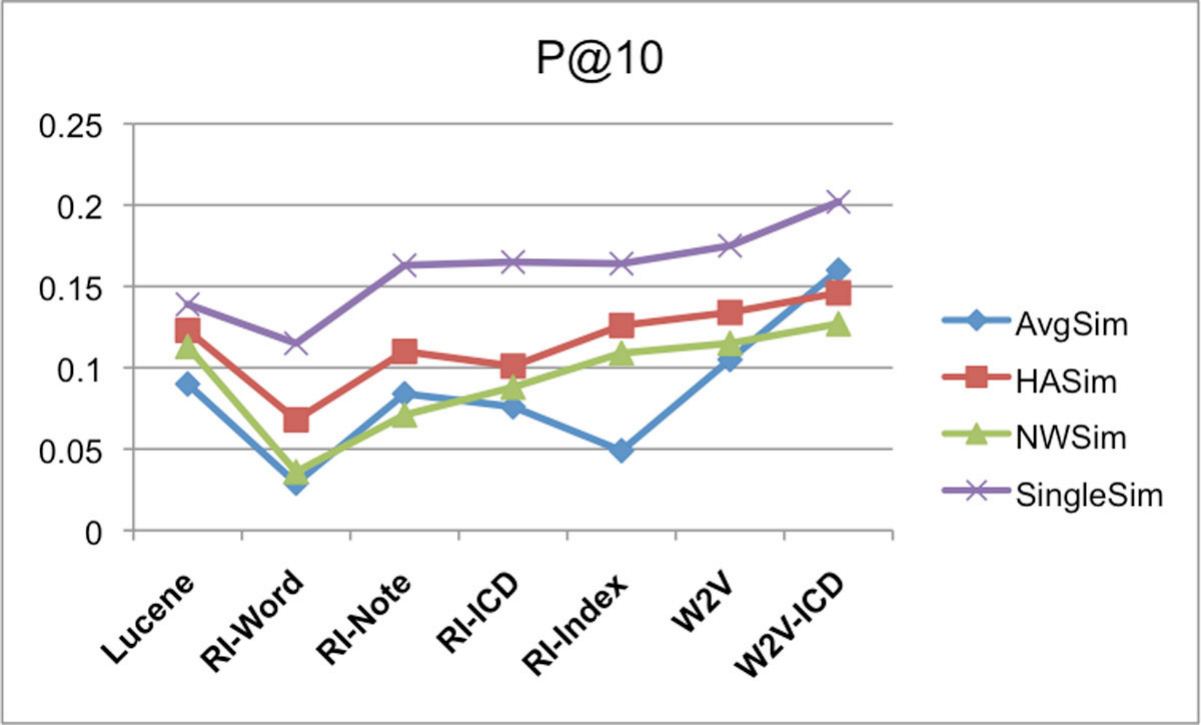
**IDFWeight-Eval - IDFWeight-Results - P@10**. Average P@10 scores for different similarity measures using the various IR models. For creating the evaluation set the IDFWeight weighting was used, and also the retrieval was done using the IDFWeight weighting.

Subsequently a 7-by-7 experimental design was followed where each retrieval method, or model, was tested against each test set. At retrieval time, for each query episode, the system retrieves and ranks 1000 care episodes. It can be expected that when identical methods are used for retrieval and test set construction, the resulting bias gives rise to relatively high scores. In contrast, averaging over the scores for all seven construction methods is expected to be a less biased estimator of performance. The way these average scores are calculated is exemplified in Table 9 for MAP scores. This is done in the same way for the other scores (Retrieved count and P@10), but not shown for reasons of space. The resulting average scores for each care episode similarity calculation approach, over the various models, are shown as follows: Retrieved counts in Figure 10, MAP in Figure 11, and P@10 are shown in Figure 12.

**Table 9:** Experiment 2: MAP scores for different IR models (rows) when using different models for measuring discharge summary similarity (columns).

Test set	Lucene	RI-Word	RI-Note	RI-ICD	RI-Index	W2V	W2V-ICD	Average	Rank
**IR model**									
**Lucene**	0.084	0.046	0.041	0.050	0.030	0.062	0.071	**0.055**	4
**RI-Word**	0.041	0.049	0.029	0.036	0.016	0.048	0.051	**0.039**	7
**RI-Note**	0.048	0.041	0.063	0.061	0.024	0.050	0.074	**0.052**	5
**RI-ICD**	0.059	0.036	0.054	0.149	0.033	0.058	0.124	**0.073**	2
**RI-Index**	0.063	0.033	0.044	0.048	0.043	0.052	0.065	**0.050**	6
**W2V**	0.075	0.051	0.052	0.079	0.035	0.094	0.106	**0.070**	3
**W2V-ICD**	0.089	0.053	0.070	0.150	0.046	0.094	0.187	**0.098**	1

This table also illustrates the general approach to how the average scores are calculated for the results graphs for Experiment 2.

The same models/systems and their parameters were used here as in Experiment 1. The Random baseline achieved the following average scores: Retrieved count = 151, MAP = 0.0004, P@10 = 0.0022.

For the results reported in Figures 10, 11 and 12, IDFWeight word weighting was used for generating both the result sets and the evaluation sets, however we also tried using the IDF*MetathesaurusWeight weighting approach when generating the result sets. When evaluated on the evaluation sets generated with IDFWeight weighting, we observed that the results for each model were typically slightly better compared to the result sets generated with IDFWeight weighting for the following models: RI-Word, RI-Note, RI-Index and W2V (average score gain +3.39%). And similar to Experiment 1, this was not the case for the RI-ICD and W2V-ICD models (average score gain *−*1.83%).

## Discussion

The goal of the experiments was to determine which distributional semantic model work best for care episode retrieval, and what the best way of calculating care episode similarity is. The experimental results show that several models outperform Lucene. This suggests that distributional semantic models contribute positively to calculating document/note similarities in the clinical domain, compared with straight forward word matching (cf. RI-Index and Lucene). Both W2V and RI-Word utilize a narrow contextual sliding window during training. The scores indicate that W2V induces a model that, among these two, is better suited for IR with the approach taken here. RI-Word did perform relatively bad, and there are reasons to believe that performance gains can be achieved through adjusting and/or optimizing the utilized weighting (cf. TF*IDF), vector normalization, and model training parameters [68,69].

The relatively good performance of the RI-ICD and W2V-ICD models suggests that exploiting structured or encoded information in building semantic models for doing clinical NLP is a promising direction that calls for further investigation. This applies to clinical NLP as well as other domains and NLP tasks. This approach concurs with the arguments in favor of reuse of existing information sources in Friedman et al. [21]. On the one hand, it may not be surprising that these models perform best in Experiment 1, given that both modelling/training and evaluation method here rely on ICD-10 codes. On the other hand, being able to accurately retrieve care episodes with similar ICD-10 codes evidently has practical value from a clinical perspective. With the evaluation used in Experiment 1, one could argue that the best performance would be achieved by a dedicated ICD-10 classification system. However, in an IR context a labeling of each care episode by a small number of ICD- 10 codes does not provide sufficient information to allow full (relative) similarity rankings of the care episodes. One would thus not be able to retrieve e.g. the top 10 most similar care episodes to a query episode in a ranked (descending) order.

Additional support for the ICD-10 code based models comes from a different evaluation strategy that makes use of the discharge summaries associated with each care episode. This method is based on the idea that similar care episodes are likely to have similar discharge summaries. Therefore an approximation of the gold standard for a query can be obtained from the top-n episodes in the collection with a summary most similar to that of the query. Notice that, same as for the ICD-10 codes, the discharge summary is not used for constructing the query. However, this approach has some drawbacks. For example, similarity between discharge summaries must be measured using the same distributional models as used in retrieval, introducing a certain amount circularity. There is also no principled way to determine the value of *n *when taking the top-n results. Yet, when using this evaluation method - which does not rely on ICD-10 codes - the ICD-based models still perform best (cf. results reported in [23]), suggesting that their good performance is not only due to the use of ICD-10 codes for evaluation purposes.

Further, the results indicates, for most models whose word vectors are built from word distribution statistics, performance gains when heightened weight is given to words matching those in a health metathesaurus. Such a list of health terms is something that can easily be obtained in most languages. The fact that RI-ICD and W2V-ICD did not benefit from such re-weighting of word vectors can be explained through how these models are trained, and that the "statistical correct" semantic meanings of words, especially in relation to the ICD-10 codes, is already induced through the training phase.

All models performed best when care episodes were viewed as atomic documents (SingleSim). Thus there seems to be no performance gain in taking the internal structure of each care episode into account, i.e., the individual clinical notes. One possible reason for this being the case would be that each note on their own, compared to all text in a full care episode, do not contain enough information to be properly discriminative for this task.

In our data a single care episode can potentially span across several hospital wards. A better correlation between the similarity measures is to be expected when using care episodes originating from a single ward. Also, taking into consideration all ICD-10 codes for care episodes - not only the primary one - could potentially improve discrimination among care episodes. This could be useful for extending the RI-ICD and W2V-ICD models by training them on the secondary ICD-10 codes as well.

Input to the models for training was limited to the free text in the clinical notes, with the exception of the use of ICD-10 codes in the RI-ICD and W2V-ICD models. Additional sources of information could, and probably should, be utilized in an actual care episode retrieval system deployed in a hospital. A prime candidate is the structured and coded information commonly found in EHR systems. Examples are patient diagnosis and treatment codes, lab test values, dates, wards visited, medications, care episode span, previous diagnosis, age, sex, classified events, and so on. Some of these may belong to an ontology or thesaurus with a certain internal structure that could be exploited, such as SNOMED CT [70] and UMLS [71] (for languages where this can be applied). Other potential sources include user- supplied keywords or information units/concepts that have been extracted from, or matched against, free text using some type of information extraction tool such as MetaMap [72]. Such structured information can be used directly for IR, or indirectly through training of models as demonstrated in the current work. One potential issue with the use of structured information is that it may be incomplete or missing, giving rise to the problem of "missing values".

## Conclusion

This paper proposes the new task of *care episode retrieval *as a special instance of information retrieval in the clinical domain. It was argued that the specialized clinical language use calls for dedicated NLP resources, which are mostly lacking - especially for languages other than English - and costly to build. Distributional models of semantics, built from a collection of raw clinical text in a fully unsu- pervised manner, were proposed as a resource-lean alternative. Several variants of *random indexing *and *word2vec *were proposed and experimentally tested. Also several approaches to calculating the overall care episode similarity on the basis of their word similarities were explored.

As manually constructing a gold standard is costly, two new evaluation strategies are introduced. One relies on the ICD-10 codes attached to care episodes, the other relies on discharge summaries. Two innovative distributional models were presented - RI-ICD and W2V-ICD - which leverage the ICD-10 codes to enhance domain- specific word similarity. These models also proved to yield best performance, out- performing a state-of-the-art search engine (Lucene). Taking the internal structure of care episodes into account, including attempts at optimal pairing or temporal alignment of individual clinical notes, did not yield any improvements.

The work presented here suggests that training a representation to associate additional knowledge to that obtained from the free text alone, such as structured domain information, is beneficial to the computation of semantic similarity. We have demonstrated how ICD-10 codes can be used indirectly for care episode retrieval, and we hypothesize that the utilized methods may also perform well when applied to more generic IR tasks within the clinical domain. Other types (structured) information units and concepts should also be explored in future work. Also, it is likely that a similar approach can be used for IR and NLP in other domains.

Our evaluation, as well as that in most of the related work, is based on pure retrieval performance. Future work on IR in the clinical domain should arguably focus more on evaluating IR-systems in terms of support for care and patient outcomes.

## Competing interests

The authors declare that they have no competing interests.

## Authors' contributions

Hans Moen (HM) was responsible for coordinating the work and was involved in all parts of it, and had the main responsibility for the manuscript. HM had the responsibility of the overall design of the study and for carrying out the experiments. HM initiated the idea of using ICD codes for training models. HM also implemented most of the utilized code.

Filip Ginter (FG) contributed to the experimental setup, writing and reviewing of the manuscript. FG did the work related to training of the W2V model. FG also initiated the idea of translating the RI-ICD principles into W2V, i.e. the W2V-ICD method, including implementing and training it.

Erwin Marsi (EM) contributed to the design of the RI models, experimental setup, data analysis, and to writing and reviewing of the manuscript. EM also contributed with supervision thoughout the work.

Laura-Maria Murtola (LM) contributed to the writing and reviewing of the manuscript. LM did also contribute with clincal domain knowledge in most aspects of the work, and provided access to the utilized metathesaurus.

Tapio Salakoski (TS) contributed through reviewing of the manuscript and supervision. TS also provided access to the utilized clinical data.

Sanna Salanterä (SS) contributed through reviewing of the manuscript and by providing access to the utilized clinical data. SS also contributed with supervision and ideas leading up to the ICD-based models.

## Declarations

Publication costs for this article were funded by the corresponding author's institution^1^.

This article has been published as part of *BMC Medical Informatics and Decision Making *Volume 15 Supplement 2, 2015: Proceedings of Louhi 2014: The Fifth International Workshop on Health Text Mining and Information Analysis. The full contents of the supplement are available online at http://www.biomedcentral.com/bmcmedinformdecismak/supplements/15/S2.
